# A Smart Eddy Current Sensor Dedicated to the Nondestructive Evaluation of Carbon Fibers Reinforced Polymers

**DOI:** 10.3390/s17091996

**Published:** 2017-08-31

**Authors:** Mohammed Naidjate, Bachir Helifa, Mouloud Feliachi, Iben-Khaldoun Lefkaier, Henning Heuer, Martin Schulze

**Affiliations:** 1 Laboratoire de Physique des Matériaux, Université de Laghouat, Laghouat 03000, Algeria; helifa@yahoo.fr (B.H.); lefkaier_ik@yahoo.fr (I.-K.L.); 2 IREENA-IUT, Université de Nantes, 44602 Saint-Nazaire, France; mouloud.feliachi@univ-nantes.fr; 3 Fraunhofer Institute for Ceramic Technologies and Systems IKTS, 01109 Dresden, Germany; henning.heuer@ikts.fraunhofer.de (H.H.); martin.schulze@ikts.fraunhofer.de (M.S.)

**Keywords:** multi-element sensor, eddy current, CFRP characterization, nondestructive testing (NDT), electromagnetic field computation, FEM modeling

## Abstract

This paper propose a new concept of an eddy current (EC) multi-element sensor for the characterization of carbon fiber-reinforced polymers (CFRP) to evaluate the orientations of plies in CFRP and the order of their stacking. The main advantage of the new sensors is the flexible parametrization by electronical switching that reduces the effort for mechanical manipulation. The sensor response was calculated and proved by 3D finite element (FE) modeling. This sensor is dedicated to nondestructive testing (NDT) and can be an alternative for conventional mechanical rotating and rectangular sensors.

## 1. Introduction

Non-destructive testing (NDT) is one of the most common and powerful techniques employed in the inspection of materials during their manufacture or use. When dealing with an electrically conductive body, eddy current non-destructive testing (EC-NDT) is the most efficient way to test the state of health of materials (low cost, readily implemented...). The efficiency of EC-NDT depends directly on the performance of the sensor used. During the last few years, researchers have focused their work to a new generation of EC probes consisting of miniaturized sensors forming a rectangular sensor array that could detect defects with high accuracy, even in complex materials such as carbon fiber-reinforced polymers (CFRP), the material studied as an application in this article. Nevertheless, the highly anisotropic and complex structure of CFRP may require, depending on the type of control to be performed, specific EC sensor configurations. For the detection of defects or the fibers’ orientation, actual solutions are based on high frequency sensors [1], rotating sensors [2,3] or rectangular sensors [4,5] These types of sensors are used to draw a polar diagram giving the intensity of the measured signal in terms of the rotation angle of the sensor. The angles of the obtained lobes determine the different fibers orientations whereas their amplitude indicates the position of the ply in the sample.

In this work, a new multi-element sensor array design is suggested with the aim of evaluating CFRP materials. This multi-element sensor is presented as an alternative to the rotating and the rectangular sensors in order to increase the sensitivity and resolution and to reduce the mechanical effort for rotation.

## 2. Conception

**Figure 1:**
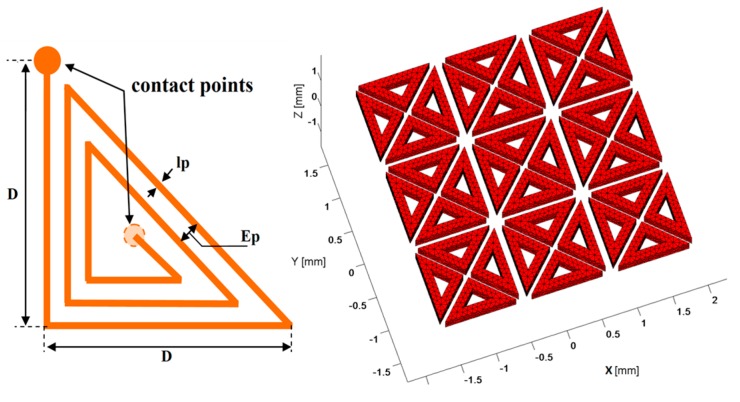
(**Left**) Design of a single element and (**Right**) the simplified array configuration.

The proposed sensor consists of flat triangular coils combined into an array. Figure 1 displays the design of the sensor’s elements as well as the simplified geometry introduced as input to the computational code for simulation.

The triangular form allows a higher flexibility on the desired shapes of the generated electromagnetic (EM) field. The elements are arranged that they can give a large number of possible EM configurations, thereby, avoid the mechanical swiveling of the sensor occurring in conventional characterization. In addition to this, such a structure can cover a relatively wide inspection area which saves the number of manual scanning operations. The size of the sensor elements can be selected to optimize precision in the defect detection.

**Figure 2:**
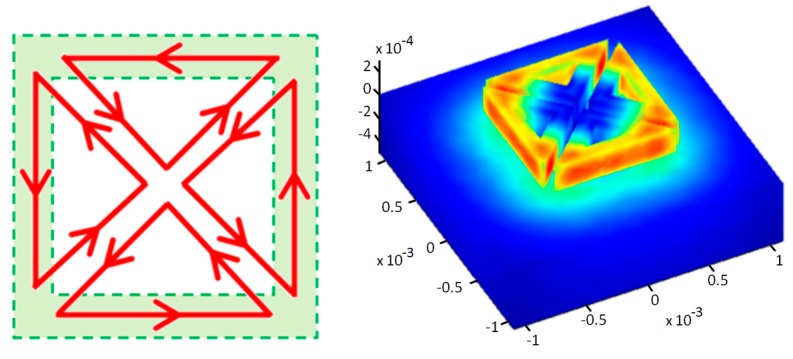
Top view of the current flow upon jointly exciting the four coils (**left**) and perspective view of the three-dimensional distribution of the magnetic potential vector for the four coils (**right**).

The basic idea rests on the fact that the EM fields generated by two parallel wires traversed by currents with the same amplitude and in opposite direction cancel each other. This property is exploited to generate different field forms, acting only on the excitation currents’ distribution. Thereby, Figure 2 represents four triangular coils excited in a way that the resulting field is similar to that obtained by a square coil: the fields generated by currents flowing in the “diagonal” conductors oppose and cancel each other. Then, the resulting field is practically that due to the currents flowing in the “external quadrature” conductors.

The mode of calculation is described hereafter.

## 3. Sensor Characteristics 

### 3.1. Geometrical Characterization

The proposed sensor is an array assembly of 36 identical coils in the shape of isosceles triangles whose angles at the base worth 45°. In each coil, the developed length or the total length of the wire *l_total_* and the total effective surface *S_total_* are given by the Equations (1) and (2) respectively (see Appendix A):

(1) $$l_{totale}\approx n\left[\left(2+\sqrt{2}\right)D-\left(n-1\right)\left(l_{p}+E_{p}\right)\left(1+\frac{2}{\tan(\pi /8)}\right)\right]$$

(2) $$S_{totale}\approx \frac{1}{2}\sum_{k=1}^{n}{\left[D-\left(k-1\right)\left(l_{p}+E_{p}\right)\left(1+\frac{1}{\tan(\pi /8)}\right)\right]}^{2}$$

where *D* is the external rib of the coil (see Figure 1), *l_p_* is the line width, *E_p_* is the inter-lines distance and *n* is the number of turns. These geometrical parameters are indispensable to calculate the electrical parameters as their influence is direct.

### 3.2. Electrical Characterization

**Figure 3:**
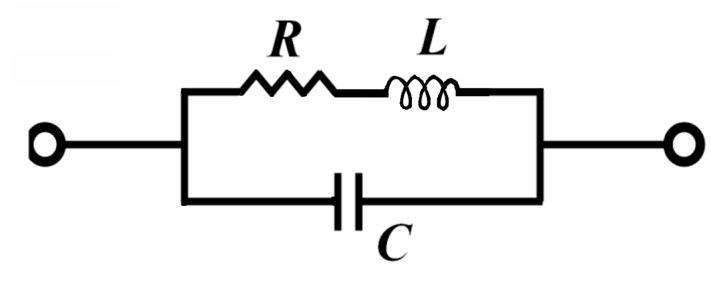
Electrical model of a coil.

The theoretical model of a coil is given in Figure 3 [6]. To determine the coil inductance *L*, and for the sake of accuracy, an evaluation of the stored magnetic energy (Equation (5)) was provided via the FE model developed in Section 4. However, to determine the resistance *R* and the capacitance *C*, basic models have been adopted to simplify the calculation. The relations through which the electrical parameters have been estimated are given below:

(3) $$R=\frac{\rho \text{×}l_{totale}}{l_{p}\text{×}h_{p}},$$

(4) $$C={\left[\sum_{k=2}^{n}\left(1/\varepsilon \frac{h_{p}\text{×}l_{k}}{E_{p}}\right)\right]}^{-1},$$

(5) $$L=\frac{\omega }{I^{2}}\underset{\Omega }{∭}\frac{1}{\mu }{\left|\vec{B}\right|}^{2}d\Omega \;,$$

where *h_p_* is the height of the line, *ρ* is its electrical resistivity of the wire, *ε* is the electric permittivity, *ω* is the angular frequency, Ω is the whole computation area (sensor and air box), *μ* is the magnetic permeability and *B* is the magnetic flux density.

### 3.3. Physical Characterization

Knowledge of its geometrical and electrical characteristics is necessary but insufficient to fully qualify the electromagnetic behaviour of a coil. As an EM sensor, the coil needs to meet other requirements depending on the intended mode of its use. As an emitter, its emissive ability must be calculated. If it’s used as receiver, it is necessary to determine its sensitivity and its electrical noise signal. In the proposed sensor, coils have the versatility to work in emission and reception simultaneously or separately, which implies a complete and rigorous study of the sensitive element. Ravat, C. [6] exposes in his work these parameters as follows:

■ According to Faraday-Lenz’s law, at a frequency *f*, the sensitivity of a coil is:
  (6) $$S=\left|\frac{dV}{dB}\right|=2\pi fS_{totale}\;,$$
  where *dV* is the voltage variation provoked by a variation in the received magnetic induction *dB*.
■ The noise of a coil when it is not carrying current is only a thermal agitation noise. This effective voltage *v_b_* at a temperature *T* and in measuring frequency range Δ*f* is given by;
  (7) $$v_{b}=\sqrt{4K\times T\times R\times \Delta f}\;,$$
  where *K* is Boltzmann’s constant.
■ The emissive ability *P_e_* is the ratio between the emitted field “*B*” and the current “*I*” necessary for its emission:
  (8) $$p_{e}=\frac{B}{I}=\frac{L}{S_{totale}}\;,$$

### 3.4. Optimization of the Coil

**Figure 4:**
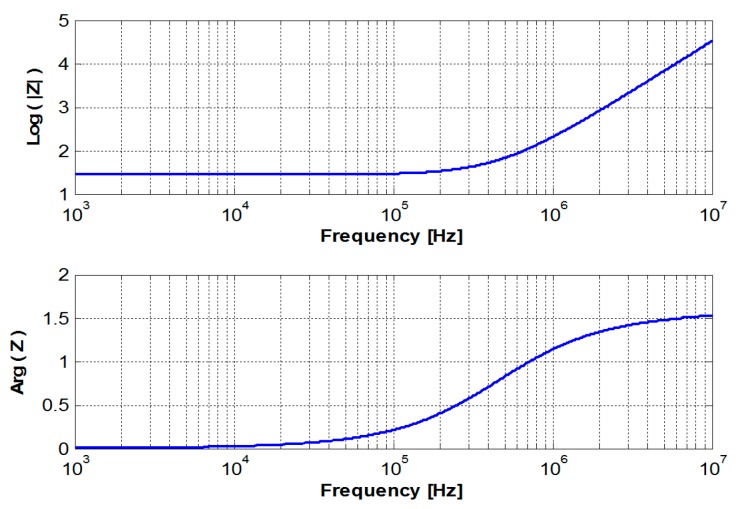
Frequency response of the sensor.

The relationship between the geometrical, electrical and physical characteristics developed previously allows us to study the influence of each parameter and thus to determine the optimum dimensions of the coil appropriate for a desired application. Table 1 provides the characteristics of the selected coil to non-destructively evaluate a CFRP. According to the theoretical model (Figure 3), Figure 4 shows the frequency response of a triangular coil using data given in Table 1. It can be seen that the coil can be used as EM field sensor above 800 kHz where it shows a strong inductive behavior with a phase greater than 60°. The cut-off frequency is much higher than 100 MHz.

## 4. Modeling

After the construction of the geometry and the mesh generation using the open-source software GMSH, the problem data are sent to our 3D finite element solver in which was implemented the magneto-dynamics formulation AV-A (Equation (9)); mathematical model chosen to describe the EM behaviour of our problem. The calculations are carried out in the harmonic regime. A penalty term is introduced to ensure the uniqueness of the solution [7]:

(9) $$\left\{ \begin{array}{c} \vec{\nabla }\times \frac{1}{\mu }\vec{\nabla }\times \vec{A}-\vec{\nabla }\left(\frac{1}{\mu }\vec{\nabla }\cdot \vec{A}\right)+\bar{\bar{\sigma }}\left(j\omega \;\vec{A}+\vec{\nabla }V\right)={\vec{J}}_{s}\\ \vec{\nabla }\cdot \left(j\omega \;\bar{\bar{\sigma }}\left(\vec{A}+\vec{\nabla }V\right)\right)=0 \end{array}\right.,$$

where $\vec{A}$ and *V* are respectively the magnetic vector potential and electric scalar potential, *μ* is the magnetic permeability and $\bar{\bar{\sigma }}$ is the electrical conductivity tensor given according to the ply orientation by [2]:

(10) $$\bar{\bar{\sigma }}=\left(\begin{array}{ccc} \sigma_{//}\cos^{2}(\theta )+\sigma_{⊥}\sin^{2}(\theta ) & \frac{\sigma_{//}-\sigma_{⊥}}{2}\sin(2\theta ) & 0\\ \frac{\sigma_{//}-\sigma_{⊥}}{2}\sin(2\theta ) & \sigma_{//}\cos^{2}(\theta )+\sigma_{⊥}\sin^{2}(\theta ) & 0\\ 0 & 0 & \sigma_{zz} \end{array}\right),$$

where *σ*_//_ is the electrical conductivity in the fibers direction, *σ*_⊥_ is the conductivity in the transverse direction of the fibers and *σ*_zz_ is the conductivity in the direction of the plies stacking.

## 5. Results

The EC-NDT concept is based on the distribution and circulation of the induced currents in the component being inspected. This distribution is strongly linked to the profile of the excitation EM field. With this in mind, numerical experiments were carried out to discern the ability of our sensor to emulate the EM field configurations obtained by conventional sensors such as a rectangular coil.

### 5.1. Sensor and EM Field

**Figure 5:**
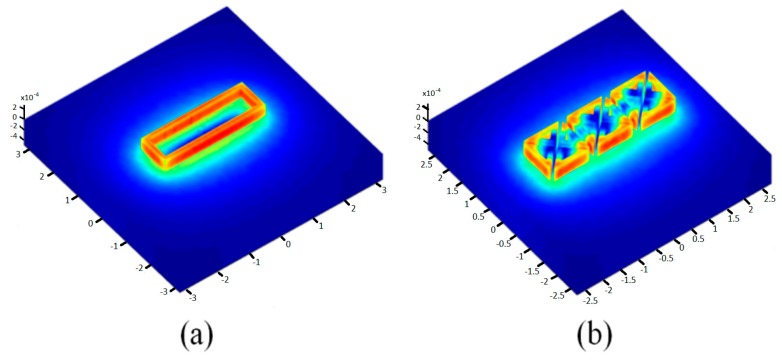
Magnetic vector potential calculated for a system of: (**a**) rectangular coil; (**b**) proposed multi-element sensor.

**Figure 6:**
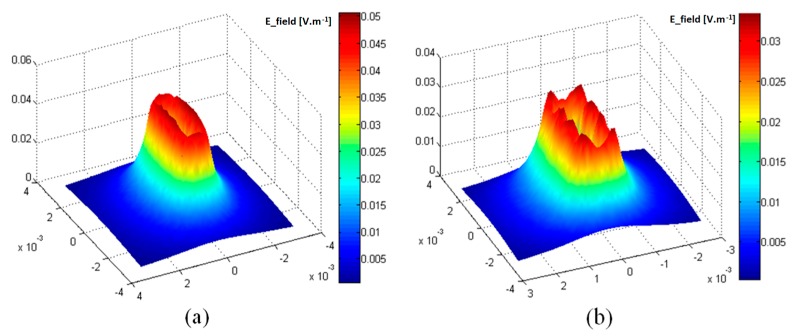
Electric field magnitude at the front surface of the load (z = 0): (**a**) rectangular coil; (**b**) multi-element sensor.

Figure 5 shows that the electromagnetic field generated by a set of triangular elements excited simultaneously is similar to the EM field created by a conventional rectangular coil. The electric field calculated at the front surface of the load illustrated in Figure 6 confirms this equivalence of the global EM behaviour for the two systems. Nevertheless, we note some electric field irregularities in the case of the multi-element sensor, which is due to the discontinuities in the geometry of the inductor and to the current singularities in the bends of triangles.

**Figure 7:**
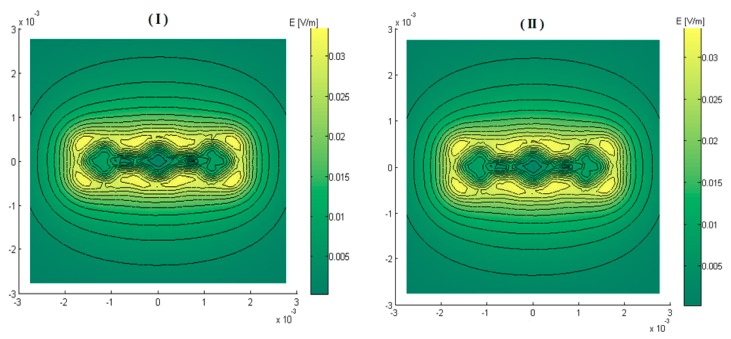
Electric field magnitude at the front surface of the load (z = 0): (I) without regard to non-excited elements (*σ* = 0); (**II**) non-excited elements are physically represented (*σ* = 30, 6 × 10^6^ S/m).

The interaction between the excited elements and those “at rest” was studied too with the aim of evaluating the coupling effect. The obtained results displayed in Figure 7 show that the non-excited adjacent elements are without a slightest action on the configuration of the field or on its amplitude at the operating frequency of 1 MHz.

**Figure 8:**
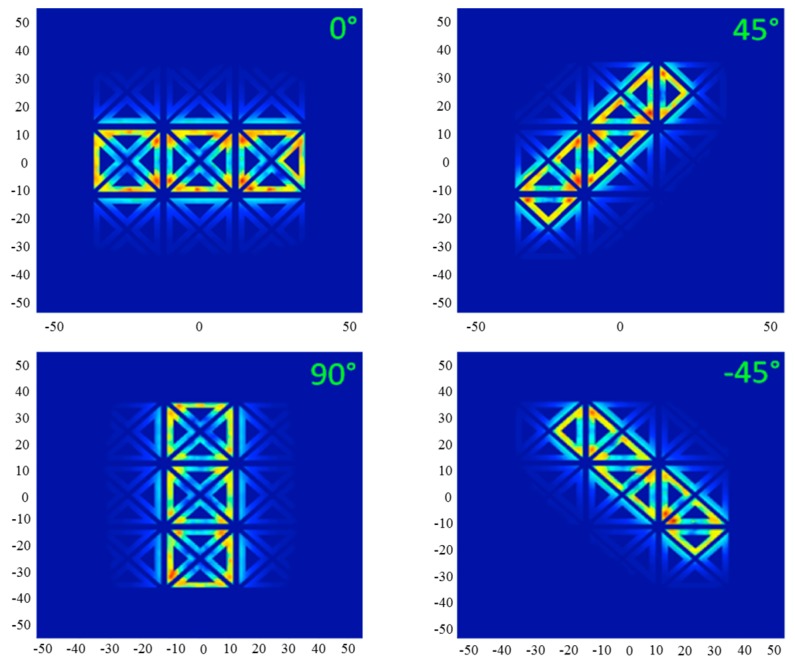
Field configurations generated by the multi-element sensor equivalent to a rectangular coil oriented at 0°, 45°, 90° and −45°.

In addition to the foregoing, the proposed sensor allows a high flexibility in terms of modes of excitation and measurement. The figure below illustrates different field configurations obtained for different excitations. It can be seen in Figure 8 that the sensor can substitute rectangular coil oriented at 0°, 45°, 90° and −45° without recurring to mechanical rotation. This property will be exploited and applied to a laminate of CFRP.

### 5.2. Application to CFRP

**Figure 9:**
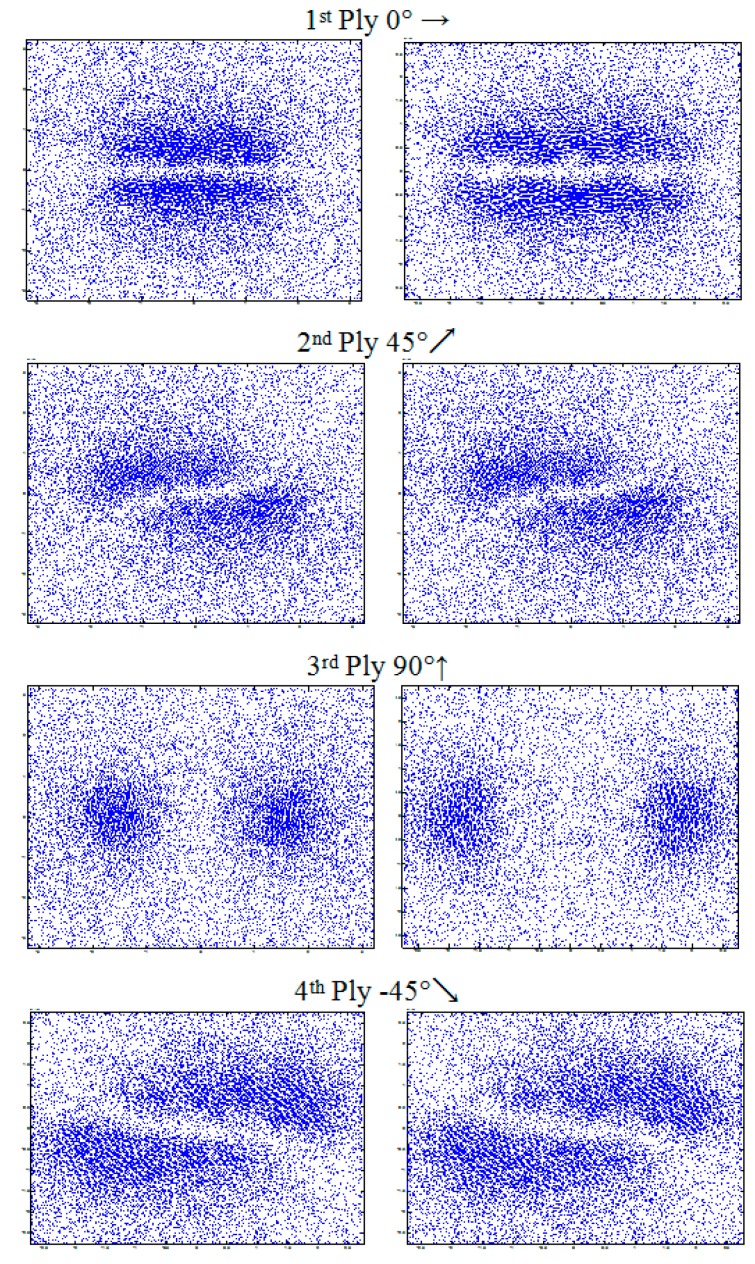
Eddy current distribution in different plies of laminate (0°, 45°, 90°, −45°); at the right caused by the multi-element sensor and at left by a rectangular coil (3.2 mm × 1 mm).

The modeled system is a stack of four plies oriented at [0°, 45°, 90°, −45°]. The physical and geometrical characteristics are given by Table 2. Figure 9 illustrates the distribution of eddy currents produced by a rectangular inductor oriented at 0° and its equivalent generated by the multi-element sensor. It is noted that the distributions of eddy currents generated by the two systems, in each ply of the laminate, is typically identical. This leads to expect, consequently, an analogue dissipated power, hence an identical response in terms of impedance.

**Figure 10:**
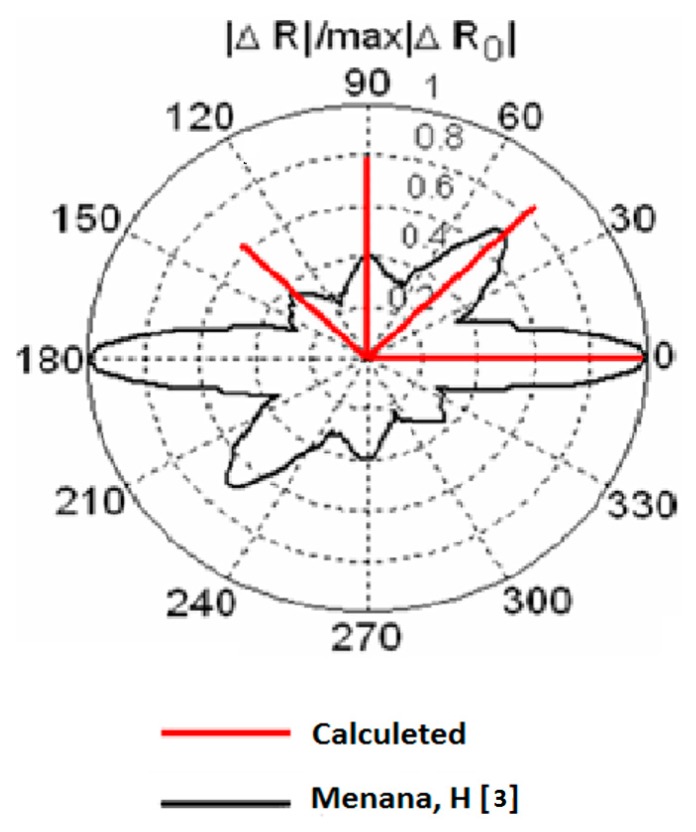
The normalized resistance of the coil as function of its rotation angle above a laminate of four plies [0°, 45°, 90°, −45°].

Furthermore, the results presented in Figure 10 prove that the proposed sensor can detect the orientation of the plies and their order of stacking. The comparison between the amplitudes of peaks shows that they are decreasing according to the stacking order of the plies. However, our values do not coincide with those calculated by [3] due to the mismatch of the two systems (number of turns and dimensions of coils). We note also that there is no large difference between the peaks at 45° and 90°; this can be explained by the change of sizes (length to width ratio) of the equivalent rectangular coil generated at 45° and 90° (see Figure 8).

## 6. Conclusions

A new design of an eddy current multi-element sensor is proposed. The EM field computation results reveal that this sensor is able to control the EM field shape and generate the plurality of configurations often needed in EC–NDT of carbon fiber-reinforced polymers. The application of the proposed sensor on a sample of CFRP shows its capability to detect the plies’ orientations and their stacking order by acting only on the excitation currents. This sensor can thus be an alternative to the mechanical rotating sensors and the rectangular sensors.

## Figures and Tables

**Table 1:** Numerical values of the coil characteristics calculated at 1 MHz.

	Parameter	Numerical Value	Unit
**Coil dimensions**	external length *D*	1	[mm]
	line width *l_p_*	6	[µm]
	inter-line space *E_p_*	3	[µm]
	number of turns *n*	33	
**Electrical parameters**	resistance *R*	4.24	[ohm]
	inductance *L*	1.44	[µH]
	capacity *C*	3.5	[fF]
	sensitivity *S*	35	[V/T]
	noise voltage *v_b_*	0.83	[µV]
	emissive ability *P_e_*	254	[mT/A]

**Table 2:** Numerical values of the system characteristics

	Parameter	Numerical Values	Unit
**Laminate**	Number of plies	4	
	Fibers orientation	0°, 45°, 90° and −45°	[°]
	Conductivity (*σ*_//_, *σ*_⊥_, *σ*_zz_)	(10^4^, 2 × 10^2^, 10)	[S/m]
	Ply thickness	125	[µm]
**Sensor**	Number of coils	36	
	Gap inter-coils	0.08	[mm]
	Lift-off	0.125	[mm]
	Current intensity	20	[mA]
	Frequency	1	[MHz]

## Appendix A

### Appendix A.1. Calculation of the Developed Length of the Coil

The coil is composed of n triangular turns (see Figure 1) numbered from the outside to the inside. These triangular turns are isosceles whose angles at the base worth 45°. The lengths of the three edges of the *k-th* turn are:

(A1) $$d_{1}\left(k\right)\approx d_{2}\left(k\right)=D-\left(k-1\right)\left(l_{p}+E_{p}\right)\left(1+\frac{1}{\mathrm{tg}\frac{\pi }{8}}\right),$$

(A2) $$d_{3}\left(k\right)\approx D\sqrt{2}-2\left(k-1\right)\left(\frac{l_{p}+E_{p}}{\mathrm{tg}\frac{\pi }{8}}\right),$$

where *d*_1_ and *d*_2_ are the identical edges, *d*_3_ is the hypotenuse.

Therefore, the developed length of a turn number *k* is:

(A3) $$l_{k}\approx d_{1}\left(k\right)+d_{2}\left(k\right)+d_{3}\left(k\right),$$

Thus, the total developed length of the entire coil is simply the sum of *l_k_*:

(A4) $$l_{totale}\approx \sum_{k=1}^{n}l_{k},$$

(A5) $$l_{totale}\approx \sum_{k=1}^{n}\left(2\left[D-\left(k-1\right)\left(l_{p}+E_{p}\right)\left(1+\frac{1}{\mathrm{tg}\frac{\pi }{8}}\right)\right]+\left[D\sqrt{2}-2\left(k-1\right)\left(\frac{l_{p}+E_{p}}{\mathrm{tg}\frac{\pi }{8}}\right)\right]\right),$$

Or more simply:

(A6) $$l_{totale}\approx n\left[\left(2+\sqrt{2}\right)D-\left(n-1\right)\left(l_{p}+E_{p}\right)\left(1+\frac{2}{\tan(\pi /8)}\right)\right],$$

### Appendix A.2. Calculation of the Total Area

The surface enclosed by the turn number *k* is:

(A7) $$S_{k}\approx \frac{1}{2}{\left[D-\left(k-1\right)\left(l_{p}+E_{p}\right)\left(1+\frac{1}{\tan(\pi /8)}\right)\right]}^{2},$$

Then, the total effective surface of the coil is:

(A8) $$S_{totale}\approx \frac{1}{2}\sum_{k=1}^{n}{\left[D-\left(k-1\right)\left(l_{p}+E_{p}\right)\left(1+\frac{1}{\tan(\pi /8)}\right)\right]}^{2},$$

## References

[1] Heuer H., Schulze M., Pooch M., Gäbler S., Nocke A., Bardl G., Cherif C., Klein M., Kupke R., Vetter R. (2015). Review on quality assurance along the CFRP value chain—Non-destructive testing of fabrics, preforms and CFRP by HF radio wave techniques. Compos. Part B Eng..

[2] Mook G., Lange R., Koeser O. (2001). Non-destructive characterization of carbon-fiber reinforced plastics by means of eddy currents. Compos. Sci. Technol..

[3] Menana H., Feliachi M. Non destructive evaluation of the conductivity tensor of a CFRP plate using a rotating eddy current sensor. Proceedings of the XIV International Symposium on Electromagnetic Fields.

[4] Savin A.R., Grimberg R., Chifan S. Evaluation of delamination in Carbon Fiber Composites Using the Eddy Current Method. Proceedings of the 15th World Conference on Non-Destructive Testing.

[5] Grimberg R., Savin A., Steigmann R., Bruma A. Eddy current examination of carbon fibres in carbon-epoxy composites and Kevlar. Proceedings of the 8th International Conference of the Slovenian Society for Non-Destructive Testing.

[6] Ravat C. (2008). Conception de Multicapteurs à Courants de Foucault et Inversion des Signaux Associés Pour le Contrôle non Destructif. Ph.D. Thesis.

[7] Helifa B. (2012). Contribution à la Simulation du CND par Courants de Foucault en vue de la Caractérisation des Fissures Débouchantes. Ph.D. Thesis.

